# Measurement properties of the Patient Health Questionnaire 15 (PHQ-15) and Somatic Symptom Disorder B-criteria scale (SSD-12), including revised 1-week versions

**DOI:** 10.1038/s41598-026-50290-y

**Published:** 2026-04-26

**Authors:** Jonna Hybelius, Sandra af Winklerfelt Hammarberg, Alice Ahnlund Hoffmann, Edward Spansk, Anna Olsson, Emma Strand, Lina Söderström Winter, Tomas Åkerlund, Daniel Björkander, Amanda Kosic, Gabriel Chahin, Majken Epstein, Erland Axelsson

**Affiliations:** 1 Division of Family Medicine and Primary Care, Department of Neurobiology, Care Sciences and Society, Karolinska Institutet, Huddinge, Sweden; 2 Liljeholmen University Primary Health Care Center, Academic Primary Health Care Center, Region Stockholm, Liljeholmstorget 7, 117 63 Stockholm, Sweden; 3 Centre for Psychiatry Research, Department of Clinical Neuroscience, Karolinska Institutet, & Stockholm Health Care Services, Region Stockholm, Stockholm, Sweden; 4 Uppsala University Hospital, Uppsala, Sweden; 5 School of Law, Psychology and Social work, Örebro University, Örebro, Sweden; 6 Division of Psychology, Department of Clinical Neuroscience, Karolinska Institutet, Stockholm, Sweden

**Keywords:** Patient health questionnaire, Psychometrics, Reliability, Surveys and questionnaires, Symptom preoccupation, Psychology, Behavioural methods

## Abstract

The Patient Health Questionnaire 15 (PHQ-15), which measures somatic symptom burden, and the Somatic Symptom Disorder B-criteria scale 12 (SSD-12), which measures symptom preoccupation, are widely used questionnaires in behavioral medicine. This study built on the existing literature by evaluating the PHQ-15 and SSD-12 in Swedish, and in revised 1-week versions that could facilitate repeated measurements. The questionnaires were completed online by clinical trial participants with persistent physical symptoms (*n* = 194), and healthy volunteers (*n* = 160). For both conventional and revised 1-week versions, we evaluated item distributions, factor structure, internal consistency, construct validity based on correlations with other constructs, and test-retest reliability. Regardless of timeframe, the PHQ-15 factor structure combined general and domain-specific factors. The SSD-12 showed a three-factor structure reflecting Expectation of a chronic course, Health anxiety, and Symptom focus and impairment. For both questionnaires, internal consistency and construct validity were mostly supported, but adequate test-retest reliability was only observed in the clinical data and for averages of timepoints. Test-retest reliability in the healthy volunteer data was poor. This study is cautiously supportive of wider use of revised PHQ-15 and SSS-12 versions with a 1-week focus to facilitate repeated measurements of somatic symptom burden and symptom preoccupation in clinical populations.

**Supplementary Information:**

The online version contains supplementary material available at 10.1038/s41598-026-50290-y.

## Introduction

The subjective experience of somatic symptoms is a major concern of medical research and practice.^1^ Somatic complaints are influenced not only by medical pathologies, but show complex, and often reciprocal, relationships with wider biological, psychological, and social factors.^2,3^ For example, a key research question in pain and fatigue research is which behavioral variables that are predictive of chronicity. To address such questions, it is necessary to develop valid methods for studying subjective somatic symptom burden, and clinically relevant responses to persistent physical symptoms.^4^.

A widespread questionnaire for measuring subjective somatic symptom burden is the Patient Health Questionnaire 15 (PHQ-15).^5^ The PHQ-15 consists of 15 items, each addressing a common somatic symptom, scored from 0 (*“not bothered at all”*) to 2 (*“bothered a lot”*), resulting in a sum score of 0–30. Most factor analyses have been indicative of a multifactor structure, with each item loading onto the combination of a domain-specific factor – typically cardiopulmonary, fatigue, gastrointestinal, or pain – and a broader, general, somatic symptom burden factor. The PHQ-15 total sum is typically found to exhibit adequate internal consistency (α = 0.81, 95% CI: 0.80–0.82; including in Swedish: 0.83, 95% CI: 0.81 to 0.85). A typical Pearson correlation with anxiety is 0.54 (95% CI: 0.49–0.59), and a typical correlation with depressive symptoms is 0.62 (95% CI: 0.56–0.67).^6^.

*Symptom preoccupation* refers to the tendency to respond strongly to, and engage in behaviors contingent on, somatic symptoms. This is a hallmark characteristic of DSM-5 somatic symptom disorder, where persistently excessive responses to somatic symptoms are present in terms of either cognitions (criterion B1), anxiety (criterion B2), or time and energy (criterion B3).^7^ The Somatic Symptom Disorder B-criteria scale 12 (SSD-12)^8^ was developed to capture the this based on twelve items scored from 0 (*“never”*) to 4 (*“very often”*), resulting in a sum of 0–48. Most factor analyses have indicated that although a tree-factor model reflective of the somatic symptom disorder *cognitive*, *affective*, and *behavioral* B criteria^7,8^ can be fitted, these factors are typically highly correlated, and a unifactorial model therefore more convincing^8–17^. A recent exploratory evaluation of a French version found support for a distinctly different three-factor solution based on 11 items. This solution specified one factor for *Health anxiety* (fear of, or preoccupation with, serious disease; items 1, 2, 4, 5; called “perceived severity” by the authors), one for *Symptom focus and impairment* (items 3, 6, 9, 10, 12; called “perceived impairment” by the authors), and one for *Expectation of a chronic course* (items 8, 11; called “expectations” by the authors)^17^. Item 7, and to some degree item 10, have shown low factor loadings in several analyses^11,13–17^. Among psychosomatic outpatients, the SSD-12 is believed to be moderately correlated with general anxiety (*r* = 0.35), and weakly correlated with depression symptoms (*r* = 0.22)^8^.

Repeated measurement schedules enable increased statistical power and improved understanding of change trajectories. The optimal frequency of assessment points may depend heavily on factors such as the clinical setting or research question, and has to balance the potential information gained against the added burden on patients or study participants^18^. In clinical research, for example in applied psychology, a week-by-week measurement schedule is relatively common. This is likely to enable high reliability in the estimation of regression lines to capture change over a measurement period of a few weeks to a few months, which is relatively common in clinical research^19^. To accomplish this, scales may have to be revised to specify a particular time frame, such as a 1-week focus. However, this assumes that such revised questionnaires maintain their beneficial measurement properties, which is far from a foregone conclusion.

The conventional PHQ-15 is phrased to concern the past 4 weeks, while the SSD-12 does not specify a time frame but is suggestive of typical functioning. We are aware of one previous study evaluating a revised 1-week version of the PHQ-15 in a Dutch convenience sample^20^. That study reported a significantly lower but still adequate internal consistency according to typical guidelines (α = 0.84 [95% CI: 0.82–0.86] vs. 0.87 [95% CI: 0.85–0.89]). A notable difference, however, was that the study participants reported significantly fewer symptoms on average when asked solely about the past week (a reduction of about one-fourth; see Fig. 1 in the cited article).^20^ Little is known about the impact of a 1-week timeframe on the SSD-12. The aim of this study was to evaluate the measurement properties of the PHQ-15 and SSD-12 when administered in Swedish, with emphasis on the relative properties of conventional and revised 1-week versions. With regard to the PHQ-15, we hypothesized that the factor structure would approximate the bifactorial model presented by Witthöft et al.^21^, and that Cronbach’s α would be approximately 0.80. With regard to the SSD-12, we hypothesized that the factor structure would approximate the 3-factor model presented by Toussaint et al.^8^, and that Cronbach’s α would be approximately 0.85–0.90. We expected the PHQ-15 and SSD-12 to exhibit a correlation of ca. 0.30–0.60 in clinical data, and a priori targets for test-retest reliability were *r* ≥ 0.70, and an intraclass correlation coefficient (ICC) ≥ 0.50, or ideally ≥ 0.70. For details, see the protocol (below) and the supplement.

## Methods

### Design

This was a psychometric study of the Patient Health Questionnaire 15 (PHQ-15), the Somatic Symptom Disorder B-criteria scale 12 (SSD-12), and their revised 1-week equivalents (PHQ-15–1W, SSD-12–1W) as administered longitudinally to individuals with persistent physical symptoms (PPS sample; *n* = 194), and healthy volunteers (*n* = 160). The PPS sample was pooled from one feasibility trial (*n* = 33/194; “SOMEX0”) and one randomized controlled trial (*n* = 161/194; “SOMEX1”) of internet-delivered exposure therapy^22,23^. These trials and the collection of healthy volunteer data were approved by the Swedish Ethical Review Authority (2020-01740, 2021-01400, 2023-06574-01). The trials were preregistered at ClinicalTrials.gov (NCT04511286, NCT04942028), and this study was preregistered in the Open Science Framework (https://osf.io/g7zdt). The clinical trials were primarily designed for the study of symptom reduction (not evaluated in the present study). The feasibility trial was designed for 80% power in tests of moderate (d = 0.50) within-group effects with 15% missing data at post-treatment, and the randomized controlled trial was designed for 80% power in tests of lower-moderate (d = 0.40) between-group effects with 20% missing data at post-treatment. The size of the healthy volunteer sample was subsequently decided to enable sufficiently robust factor analyses in light of simulation studies indicating approximately 300 participants often being a reasonable target^24–26^. The project was based at Liljeholmen University Primary Health Care Center in collaboration with Karolinska Institutet, Stockholm, Sweden. The study procedures adhered to the declaration of Helsinki and its amendments, and all participants provided informed consent. Reporting follows COSMIN^27^.

### Recruitment

In the recruitment of PPS participants, the feasibility trial relied solely on online self-referrals, while the randomized controlled trial combined online self-referrals with routine care referrals to the primary care clinic. Both trials were advertised primarily via social media. Applicants completed a standardized set of questionnaires pertaining primarily to sociodemographic and clinical characteristics. This was followed by an eligibility interview with a psychologist or psychotherapist, who worked under the supervision of the first and last author. Both trials recruited participants with persistent physical symptoms, though the main eligibility criterion differed slightly. In the feasibility trial, all participants were required to meet full criteria for DSM-5 somatic symptom disorder, which requires a pattern of excessive or disproportionate cognitions, emotions, or overt behaviors related to physical symptoms.^7^ In the randomized controlled trial, a broader and more pragmatic criterion was used: participants were required to be either much bothered by at least one somatic symptom (2 points on at least one PHQ-15 item^28,29^ or exhibit at least a moderate overall somatic symptom burden (PHQ-15 sum ≥ 10^30,31^), with distress related to somatic symptoms ≥ 4 months. In both trials, participants were ≥ 18 years old and fluent in Swedish. Although comorbidities were allowed, symptoms were required not to be best explained by, and the clinical picture not dominated by, principal pathological health anxiety (hypochondriasis)^32^ or a non-somatoform psychiatric disorder such as depression, panic disorder, or a chronic stress disorder. Applicants were also excluded if medically unfit for, or undergoing medical procedures problematic for, exposure treatment. Healthy volunteers were recruited via social media advertisements and participated in exchange for a gift card. This sample was matched to the PPS sample based on age and gender, and applied via the same web platform, followed by an eligibility interview with a psychologist. All volunteers were ≥ 18 years old and fluent in Swedish. Exclusion criteria were a psychiatric disorder or a score of 2 (*“bothered a lot”*) on any item of the PHQ-15.

### Instruments

#### Structured interview schedules for diagnostic assessment

The structured eligibility interviews were aided by the Mini-International Neuropsychiatric Interview (MINI)^33^ and the Preoccupation Diagnostic Interview (HPDI; OSF.io identifier ydmcf)^34^.

#### Administration of questionnaires, and measurement schedule

We administered all questionnaires via a simple web-based interface with black text on white background, and responses given via radio buttons; accessible using any web-enabled device. The PPS sample completed the conventional PHQ-15 and SSD-12 versions at screening only, and the revised 1-week versions at subsequent timepoints: pre-treatment, after each week in treatment, and post-treatment. The healthy volunteers completed both versions at screening, and again 14–18 days later (M = 14.7, SD = 1.0). Unweighted sum scoring was employed, i.e., without multiplication by item weights, to reflect typical use of the scales.

#### Questionnaires of primary interest

The Patient Health Questionnaire 15 (PHQ-15) is a 15-item measure of somatic symptom burden^5^, typically found to reflect a combination of symptom domain-specific factors (cardiopulmonary, fatigue, gastrointestinal, pain) and a general symptom burden factor, but with redundant items^6^. The Somatic Symptom Disorder B-criteria scale 12 (SSD-12) is a 12-item measure of symptom preoccupation as conceptualized in the somatic symptom disorder B criteria^8^, typically found to be best conceptualized as unifactorial^8–17^, but often with low factor loadings for item 7^11,13–17^. See the introduction.

#### Other questionnaires, for the assessment of construct validity

To enable the assessment of construct validity, we report correlations with the following questionnaires: the Patient Health Questionnaire 2 (PHQ-2) as a measure of core depression symptoms^35^, the GAD-7 as a measure of general anxiety^36^, the 14-item Health Anxiety Inventory (HAI-14) as a measure of health anxiety^37^, and the 12-item self-report World Health Organization Disability Assessment Schedule 2.0 as a measure of disability (WD2-12)^38^.

### Randomization and clinical interventions

All PPS participants in the feasibility trial were enrolled in exposure therapy (*n* = 33). PPS participants in the randomized controlled trial were randomized 1:1 to either exposure therapy (*n* = 80) or healthy lifestyle promotion (*n* = 81). All interventions were delivered in a linear therapist-guided text format. The exposure therapy was designed to be transdiagnostic, and was tailored to the physical symptoms and responses to symptoms deemed relevant by each participant. Participants were encouraged to engage in daily exposure exercises and to refrain from strategies aimed at reducing short-term distress, to achieve therapeutic effects. The healthy lifestyle promotion involved support from a clinician to implement lifestyle changes commonly recommended for individuals with persistent physical symptoms and for primary care patients in general. This protocol emphasized physical activity, a healthy diet, and introduced rudimentary stress management techniques. For more information about the interventions, see the primary publication supplement.^23^.

### Statistical analysis

All measurement properties were first evaluated for the conventional PHQ-15 and SSD-12 versions, and then for the revised 1-week versions. Descriptive data were tabulated in Jamovi 2.3.28, including adjusted item-total correlations (ITCs) with a target of ≥ 0.50. Skewness and kurtosis were assessed, with 0 indicating no skewness or deviation in kurtosis, and absolute skewness values > 2 commonly regarded noteworthy^39^. In R 4.4.2 with lavaan 0.6–19, confirmatory factor analyses were then based on means and variance adjusted weighted least squares (WLSMV) estimation, which is robust to skewed distributions and items with few response options^40^. These analyses focused on the pooled sample with a size of 354; acceptable under most scenarios^24–26^. In sensitivity analyses, we refitted the models on the PPS sample data only. Robust fit indices were used, with typical rule-of-thumb criteria for adequate model fit in terms of a Comparative Fit Index (CFI) ≥ 0.95, Tucker-Lewis Index (TLI) ≥ 0.95, and a Root Mean Square Error of Approximation (RMSEA) < 0.08 or ideally < 0.06^41^. Modification indices were reviewed for misspecification and justifiable improvements. We evaluated internal consistency based on α and ω. We also calculated Pearson correlations versus constructs relevant for construct validity: depression symptoms, general anxiety, health anxiety, and disability. Two-week test-retest reliability was evaluated based on Pearson correlations and the two-way random effects absolute agreement ICC using the healthy volunteer data. We also estimated a lower bound for reliability in the PPS data by comparing the pre-treatment assessment to week 2 in therapy. Although we originally intended to develop algorithms to convert scores between scale versions, because reliability was questionable for the healthy volunteers (see below) and the two versions were never completed on the same day by the PPS participants, we decided not to pursue this further. In this study, absolute values for Pearson correlations of 0.10 are interpreted as weak, 0.30 as moderate, and 0.50 as strong^42^.

## Results

### Flow of recruitment

In recruitment of the PPS participants, 407 out of 601 trial applicants (68%) either withdrew their application (143/601; 24%), were excluded (235/601; 39%), or could not be reached (29/601; 5%). The most common reason for exclusions was a medical condition or ongoing treatment that made exposure therapy inappropriate (99/601; 16%), followed by a non-somatoform disorder or health anxiety disorder being the primary clinical problem (50/601; 8%). See the primary publications of each clinical trial for details^22,23^. In recruitment of the healthy volunteers, 21 out of 181 study applicants (12%) either withdrew their application (1/181; 1%), were excluded (11/181; 6%), or could not be reached (9/181; 5%). The most common reason for exclusions was clinically significant physical symptoms (6/181; 3%), followed by a neurodevelopmental or other psychiatric disorder (5/181; 3%).

### Sample characteristics

The PPS and healthy samples both had a mean age of 48 (SD = 12 vs. 13). A clear majority were women (82% vs. 85%) and had a postsecondary education (81% vs. 86%). The five most common non-DSM conditions in the PPS sample were irritable bowel syndrome (59/161), exhaustion disorder (54/161; a burnout-like chronic stress disorder^43^, osteoarthritis (49/161), hypertension (40/161), and asthma (35/161). See Table 1.

**Table 1:** Participant characteristics.

	Patients with PPS (*n* = 194)	Healthy volunteers (*n* = 160)
*Sociodemographics*		
Age, M (SD), median (range)	47.7 (12.4), 49 (20–73)	47.8 (12.6), 50 (18–75)
Female, n (%)	159 (82%)	136 (85%)
Postsecondary education, n (%)	157 (81%)	138 (86%)
Married or de facto, n (%)	136 (70%)	117 (73%)
Parent, n (%)	139 (72%)	109 (68%)
Occupation, n (%)		
Employed	153 (79%)	121 (76%)
Retired	12 (6%)	16 (10%)
Student	9 (5%)	13 (8%)
Unemployed	9 (5%)	6 (4%)
Disability pension	5 (3%)	0 (0%)
Other or unclear	6 (3%)	4 (3%)
*Clinical domains*,* M (SD)*,* median (range)*		
Somatic symptom burden (PHQ-15)	12.8 (4.8), 12.5 (2–30)	2.5 (2.3), 2 (0–12)
Symptom preoccupation (SSD-12)	27.4 (8.3), 27 (8–47)	2.8 (3.6), 1 (0–15)
General anxiety (GAD-7)	6.6 (4.7), 6 (0–21)	1.0 (1.7), 0 (0–9)
Depression core symptoms (PHQ-2)	2.0 (1.8), 2 (0–6)	0.2 (0.6), 0 (0–4)
Disability (WD2-12)	26.8 (17.2), 24.0 (0-71.8)	2.1 (4.9), 0 (0-31.3)

PHQ-2, Patient Health Questionnaire 2; PHQ-15, Patient Health Questionnaire 15 (conventional version); PPS, persistent physical symptoms; SSD-12, Somatic Symptom Disorder B-criteria scale 12 (conventional version); WD2-12, 12-item self-report World Health Organization Disability Assessment Schedule 2.

### Patient Health Questionnaire 15 (PHQ-15): original and 1-week versions

#### PHQ-15 item distributions and correlations

The most endorsed PHQ-15 item was 14 (tired or low energy). Item 8 (fainting spells) showed excess skewness. Three items had ITCs below 0.50: item 4 (menstrual problems; 0.36), item 8 (fainting spells; 0.37), and item 11 (sexual pain/problems; 0.34). Items from the 1-week version showed similar distributions and correlations, but with items 4, 6 and 11 also exhibiting excess skewness, and item 4 exhibiting an even lower ITC of 0.22. See Table 2. For the healthy volunteers, who completed both scale versions on the same day, the sum was 2.54 (SD = 2.25) for the conventional version and 2.27 (SD = 2.05) for the 1-week version.

**Table 2:** Baseline item characteristics.

Item, abbreviated	Conventional version						1-week version					
	Mean	Median	SD	Skewness	Kurtosis	ITC	Mean	Median	SD	Skewness	Kurtosis	ITC
PHQ-15 (each scored 0–2)												
1. Stomach pain	0.67	0	0.78	0.65	-1.06	0.66	0.59	0	0.75	0.83	-0.74	0.58
2. Back pain	0.67	0	0.77	0.64	-1.03	0.60	0.62	0	0.71	0.71	-0.74	0.49
3. Pain arms, legs, joints	0.82	1	0.82	0.35	-1.42	0.51	0.75	1	0.77	0.45	-1.17	0.54
4. Menstrual problems	0.30	0	0.60	1.87	2.28	0.36	0.22	0	0.51	2.25	4.20	0.22
5. Headaches	0.63	1	0.69	0.64	-0.72	0.50	0.56	0	0.65	0.72	-0.51	0.50
6. Chest pain	0.25	0	0.52	1.98	3.03	0.47	0.23	0	0.48	2.02	3.35	0.49
7. Dizziness	0.38	0	0.59	1.28	0.62	0.59	0.33	0	0.57	1.57	1.44	0.55
8. Fainting spells	0.09	0	0.30	3.53	12.64	0.37	0.03	0	0.18	5.17	24.90	0.34
9. Palpitations	0.48	0	0.71	1.15	-0.09	0.56	0.36	0	0.60	1.45	1.03	0.54
10. Short breath	0.35	0	0.57	1.39	0.96	0.57	0.33	0	0.57	1.53	1.35	0.57
11. Sexual pain/problems	0.23	0	0.55	2.31	4.16	0.34	0.16	0	0.46	2.99	8.13	0.30
12. Constipation, diarrhea	0.76	1	0.80	0.46	-1.27	0.59	0.68	0	0.78	0.63	-1.07	0.64
13. Nausea, gas, dyspepsia	0.76	1	0.83	0.47	-1.41	0.69	0.68	0	0.79	0.63	-1.11	0.65
14. Tired or low energy	1.00	1	0.82	-0.01	-1.52	0.74	0.93	1	0.81	0.14	-1.48	0.74
15. Trouble sleeping	0.76	1	0.77	0.44	-1.17	0.61	0.68	1	0.73	0.57	-0.94	0.58
*Sum of all 15 items*	8.15	7	6.39				7.15	6	5.76			
SSD-12 (each scored 0–4)												
1. Symptoms sign of disease	1.22	1	1.16	0.65	-0.46	0.74	1.02	1	1.15	0.96	0.04	0.79
2. Worried about health	1.43	1	1.28	0.49	-0.90	0.85	1.25	1	1.27	0.63	-0.71	0.90
3. Worrying is an obstacle	1.12	1	1.29	0.85	-0.49	0.84	0.94	0	1.17	1.02	-0.03	0.83
4. Convinced symptoms serious	1.03	1	1.19	1.03	0.10	0.80	0.89	0	1.14	1.15	0.43	0.79
5. Scared by symptoms	1.27	1	1.35	0.64	-0.88	0.86	1.13	1	1.26	0.73	-0.69	0.88
6. Preoccupied most of the day	1.38	1	1.46	0.51	-1.19	0.86	1.20	1	1.38	0.71	-0.87	0.82
7. Reassured by others	1.00	0.5	1.22	0.96	-0.20	0.57	0.88	0	1.17	1.11	0.14	0.61
8. Symptoms will not disappear	1.83	2	1.64	0.10	-1.64	0.86	1.70	2	1.55	0.20	-1.52	0.88
9. Worrying takes energy	1.44	1	1.49	0.44	-1.31	0.92	1.29	1	1.40	0.60	-1.04	0.88
10. Doctor does not seriously	1.17	1	1.29	0.77	-0.58	0.73	1.03	0.5	1.21	0.83	-0.47	0.73
11. Symptoms will persist	1.97	2	1.60	-0.04	-1.60	0.86	1.83	2	1.55	0.06	-1.55	0.87
12. Focusing on other things	1.42	1	1.45	0.48	-1.18	0.88	1.25	1	1.32	0.55	-1.00	0.85
*Sum of all 12 items*	16.28	17	13.96				14.39	14	12.28			

Estimates for the pooled sample (*N* = 354). Note that in the clinical subsample, the conventional and 1-week versions were not administered on the same day. ITC, adjusted item-total correlation; PHQ-15, Patient Health Questionnaire 15; SSD-12, Somatic Symptom Disorder B-criteria scale 12.

#### PHQ-15 factor analysis

As is illustrated in Table 3, none of the planned factor analyses were indicative of acceptable fit. The unifactorial model performed the worst, and the remaining models fared about equally poorly. Subsequent evaluation of the factor structure was exploratory, and did not follow a preregistered plan. Ultimately, we could retain only 11 items and found support for a 3-factor solution, which could be fitted both as a 2-tier and bifactor variant (Table 3). The local factors were: *Cardiopulmonary* (items 6, 7, 9, 10), *Gastrointestinal* (items 1, 12, 13), and *Pain/fatigue* (items 2, 3, 14, 15). In the pooled data, the average variance extracted by the general factor was 50.4% for the 2-tier variant, and 50.1% for the bifactor variant. For the 1-week revised version items, fit indices were similar but only the 2-tier (not bifactor) variant was stable. The average variance extracted by the general factor was 49.5%. In the following text, the conventional PHQ-15 is referred to as “PHQ-15”, the 11 items retained are referred to as “PHQ-11”, and 1-week versions have the suffix “1 W”. For details, see page 4 of the supplement and factor loadings in Tables S3 and S4.

**Table 3:** Factor analyses of the Patient Health Questionnaire 15 (PHQ-15).

Model		Dataset	Conventional version, robust fit indices					1-week version, robust fit indices				
Description	Factors		c2	df	CFI	TLI	RMSEA (90% CI)	c2	df	CFI	TLI	RMSEA (90% CI)
*Planned factor analyses based on 13 items (without items 4 [menstrual problems] and 8 [fainting spells])*												
1-factor	1	Pooled (primary)	332.9	65	0.77	0.72	0.18 (0.16, 0.20)	323.0	65	0.73	0.68	0.19 (0.17, 0.22)
		PPS sample only	353.4	65	0.40	0.28	0.20 (0.17, 0.22)	310.3	65	0.45	0.34	0.19 (0.17, 0.22)
3-factor, 2-tier	3 + 1	Pooled (primary)	147.2	62	0.92	0.90	0.11 (0.08, 0.14)	124.9	62	0.89	0.86	0.13 (0.10, 0.16)
		PPS sample only	162.4	62	0.81	0.76	0.11 (0.09, 0.14)	137.5	62	0.78	0.73	0.12 (0.10, 0.15)
4-factor, 1-tier	4	Pooled (primary)	132.7	59	0.93	0.91	0.11 (0.08, 0.13)	117.6	59	0.89	0.85	0.13 (0.10, 0.16)
		PPS sample only	142.1	59	0.87	0.82	0.10 (0.07, 0.13)	129.7	59	0.79	0.72	0.13 (0.10, 0.15)
4-factor, 2-tier	4 + 1	Pooled (primary)	130.4	61	0.93	0.91	0.10 (0.08, 0.13)	p.w.c.^a^				
		PPS sample only	136.5	61	0.87	0.83	0.10 (0.07, 0.13)	p.w.c.^a^				
*Non-planned factor analyses based on 12 items (without item 11 [sexual pain/problems])*												
3-factor, 2-tier	3 + 1	Pooled (primary)	115.0	51	0.93	0.91	0.11 (0.08, 0.14)	105.7	51	0.92	0.90	0.12 (0.09, 0.14)
		PPS sample only	114.8	51	0.85	0.80	0.11 (0.08, 0.14)	107.4	51	0.83	0.78	0.12 (0.09, 0.15)
4-factor, 1-tier	4	Pooled (primary)	98.4	48	0.95	0.93	0.10 (0.07, 0.13)	97.6	48	0.92	0.90	0.12 (0.09, 0.15)
		PPS sample only	92.4	48	0.90	0.87	0.09 (0.05, 0.12)	97.3	48	0.83	0.77	0.12 (0.09, 0.15)
4-factor, 2-tier	4 + 1	Pooled (primary)	96.3	50	0.95	0.93	0.10 (0.07, 0.13)	p.w.c.^a^				
		PPS sample only	88.9	50	0.91	0.88	0.09 (0.05, 0.12)	p.w.c.^a^				
*Non-planned factor analyses based on 11 items (without item 5 [headaches])*												
3-factor, 2-tier	3 + 1	Pooled (primary)	101.1	41	0.94	0.91	0.11 (0.08, 0.15)	87.0	41	0.94	0.92	0.11 (0.08, 0.14)
		PPS sample only	102.7	41	0.85	0.80	0.12 (0.08, 0.15)	91.5	41	0.85	0.80	0.12 (0.08, 0.15)
4-factor, 1-tier	4	Pooled (primary)	62.4	38	0.97	0.96	0.08 (0.04, 0.12)	60.5	38	0.96	0.94	0.09 (0.06, 0.13)
		PPS sample only	p.w.c.^b^					53.2	38	0.91	0.86	0.10 (0.06, 0.13)
4-factor, 2-tier	4 + 1	Pooled (primary)	63.0	40	0.97	0.96	0.08 (0.04, 0.12)	p.w.c.^a^				
		PPS sample only	p.w.c.^b^					p.w.c.^a^				
*Non-planned factor analyses based on 11 items*,* with covariance over items 2 (back pain) and 3 (pain arms*,* legs*,* joints)*												
3-factor, 2-tier	3 + 1	Pooled (primary)	60.0	40	0.97	0.96	0.08 (0.03, 0.12)	59.9	40	0.96	0.94	0.09 (0.05, 0.13)
		PPS sample only	58.7	40	0.95	0.94	0.07 (0.00, 0.11)	62.3	40	0.90	0.86	0.10 (0.06, 0.13)
3-factor, bifactor	3 + 1	Pooled (primary)	38.1	32	0.99	0.99	0.04 (0.00, 0.10)	40.1	32	0.99	0.97	0.06 (0.00, 0.11)
		PPS sample only	36.1	32	1.00	1.00	0.00 (0.00, 0.09)	p.w.c.^b, c^				

The pooled sample included 354 participants, of whom 194 were clinical trial participants with persistent physical symptoms, and 160 healthy volunteers. All models were fitted using weighted least square mean and variance adjusted (WLSMV) estimation, with robust fit indices. Bifactor models with 3–4 symptom domain factors were also evaluated using 11–13 items and without covariance over items 2 and 3, all of which either did not converge or had solutions with negative variances (not tabulated). Evaluating the 4-factor models in the final step with covariance over items 2 and 3 was not relevant because these two items constituted the entire pain subfactor in the previous 11-item step. p.w.c., problems with convergence and/or Heywood cases; PPS, persistent physical symptoms.

^a^ Negative variance for the fatigue factor.

^b^ Negative variance for item 2 (back pain).

^c^ Negative variance for item 3 (pain arms, legs, joints).

#### PHQ-15 internal consistency

As is reported in Table 4, the PHQ-15, PHQ-11, and all three PHQ-11 subscales exhibited adequate internal consistency in the pooled data. In the PPS subsample, only the PHQ-15 and the PHQ-11 gastrointestinal subscale exhibited an α and ω ≥ 0.70. Results were similar for the PHQ-15–1W and PHQ-11–1W.

**Table 4:** Internal consistency of total and subscale sum scores.

		Conventional scale versions				1-week revised versions			
	Items in sum	Pooled sample (*N* = 354)		PPS sample (*n* = 194)		Pooled sample (*N* = 354)		PPS sample (*n* = 194)	
		α	ω	α	ω	α	ω	α	ω
PHQ-15	15	0.88	0.88	0.72	0.73	0.87	0.88	0.72	0.71
PHQ-11	11	0.88	0.88	0.68	0.59	0.87	0.87	0.69	0.60
PHQ-11-CP	4	0.76	0.76	0.64	0.65	0.73	0.73	0.60	0.60
PHQ-11-GI	3	0.86	0.86	0.78	0.79	0.86	0.87	0.79	0.79
PHQ-11-P/F	4	0.81	0.81	0.62	0.63	0.78	0.78	0.59	0.59
SSD-12	12	0.96	0.96	0.86	0.86	0.96	0.97	0.88	0.88
SSD-8	8	0.96	0.96	0.83	0.83	0.96	0.96	0.87	0.87
SSD-8-EC	2	0.96	N/A	0.83	N/A	0.96	N/A	0.88	N/A
SSD-8-FI	3	0.93	0.93	0.77	0.78	0.91	0.91	0.73	0.73
SSD-8-HA	3	0.90	0.91	0.79	0.80	0.94	0.94	0.87	0.87

N/A, not applicable due to only 2 items. PPS, persistent physical symptoms. PHQ-15/11, Patient Health Questionnaire 15 and 11-item version – subscales being: CP, cardiopulmonary symptoms; GI, gastrointestinal symptoms; P/F, pain and fatigue symptoms. SSD-12/8, Somatic Symptom Disorder B-criteria scale 12 and 8-item version – subscales being: EC, expectation of a chronic course; FI, symptom focus and impairment; HA, health anxiety.

#### PHQ-15 correlations relevant for construct validity, including change scores

Correlations with other constructs are reported in Tables S7 and S8. In the two subsamples, correlations with the SSD-12 were moderate (*r* = 0.30 for the PPS subsample, *r* = 0.35 for the healthy volunteers). In the pooled data, correlations with general anxiety and depression were strong (*r* = 0.64, 0.60). Similar results were seen for the PHQ-15–1W (see Table S1). In the PPS subsample, the correlation between change in the PHQ-15–1W and change in the SSD-12–1W (i.e., fitted regression lines over the treatment phase) was strong (*r* = 0.52).

#### PHQ-15 test-retest reliability

The conventional PHQ-15 was administered twice within 14.7 (SD = 0.9) days in the healthy volunteer data, where test-retest reliability was below target (ICC = 0.61, *r* = 0.63). The PHQ-15–1W had poor test-retest reliability in the healthy volunteer data (ICC = 0.58, *r* = 0.58), but not as an average over two timepoints (ICC = 0.74). In the PPS data, measurements could only be compared over the first 16.3 (SD = 1.7) days of treatment. Despite the ongoing intervention, reliability was borderline acceptable (ICC = 0.63, *r* = 0.67) and clearly acceptable as an average over two timepoints (ICC = 0.77). For details, see Table S9.

### Somatic symptom disorder B-criteria scale 12 (SSD-12): original and 1-week versions

#### SSD-12 item distributions and correlations

The most endorsed SSD-12 item was item 11 (symptoms will persist). For both the original and 1-week versions, all SSD-12 items had skewness values of 2 or lower, and ITCs of at least 0.50. The lowest ITCs were exhibited by item 7 (0.57 for the original version, 0.61 for the 1-week version). See Table 2.

#### SSD-12 factor analysis

As illustrated in Table 5, neither the most widespread 3-factor model nor the unifactorial model achieved adequate fit. Subsequent evaluation of the factor structure was exploratory, and did not follow a preregistered plan. Ultimately, we found that the recently proposed solution by Pignon and colleagues^17^ fared considerably better, especially with the addition of covariance parameters and when only the most promising 8 items were retained. The factors were: *Expectation of a chronic course* (items 8, 11), *Health anxiety* (items 1, 2, 5), and *Symptom focus and impairment* (items 3, 6, 9). Correlations were: *Expectation of a chronic course* vs. *Health anxiety* 0.85, *Expectation of a chronic course* vs. *Symptom focus and impairment* 0.92, and *Health anxiet*y vs. *Symptom focus and impairment* 0.94. For the 1-week revised version items, fit indices were similar but slightly less favorable. In the following text, the conventional SSD-12 is referred to as “SSD-12”, the 8 items retained are referred to as “SSD-8”, and the 1-week versions are given the suffix “1 W”. For details, see page 6 of the supplement and factor loadings in Tables S5 and S6.

**Table 5:** Factor analyses of the Somatic Symptom Disorder B-criteria scale 12 (SSD-12).

Model		Dataset	Conventional version, robust fit indices					1-week version, robust fit indices				
Description	Factors		c2	df	CFI	TLI	RMSEA (90% CI)	c2	df	CFI	TLI	RMSEA (90% CI)
*Planned factor analyses*,* based on 12 items*												
1-factor	1	Pooled (primary)	579.1	54	0.83	0.80	0.23 (0.21, 0.26)	559.6	54	0.81	0.77	0.26 (0.23, 0.28)
		PPS sample only	547.5	54	0.61	0.52	0.22 (0.20, 0.25)	482.3	54	0.58	0.48	0.25 (0.23, 0.28)
3-factor “SSD B criteria”, 2-tier	3 + 1	Pooled (primary)	448.7	51	0.86	0.82	0.22 (0.20, 0.25)	p.w.c.^a^				
		PPS sample only	405.3	51	0.68	0.59	0.21 (0.19, 0.23)	p.w.c.^a^				
*Non-planned factor analyses based on 11 items (without item 7 [reassured by others])*												
1-factor	1	Pooled (primary)	540.8	44	0.83	0.79	0.25 (0.23, 0.28)	510.0	44	0.81	0.76	0.28 (0.25, 0.31)
		PPS sample only	520.3	44	0.62	0.52	0.24 (0.22, 0.27)	439.2	44	0.57	0.46	0.27 (0.25, 0.30)
3-factor “SSD B criteria”, 2-tier	3 + 1	Pooled (primary)	415.2	41	0.86	0.81	0.24 (0.22, 0.27)	p.w.c.^b^				
		PPS sample only	380.5	41	0.70	0.60	0.22 (0.19, 0.25)	p.w.c.^b^				
3-factor “Pignon et al.”, 1-tier	3	Pooled (primary)	216.0	41	0.96	0.95	0.12 (0.10, 0.15)	262.4	41	0.94	0.92	0.16 (0.13, 0.18)
		PPS sample only	132.7	41	0.93	0.90	0.11 (0.08, 0.14)	180.9	41	0.89	0.85	0.15 (0.12, 0.17)
*Also with covariance over items 1 (symptoms sign of disease) and 4 (convinced symptoms serious)*,* and 6 (preoccupied most of the day) and 12 (focusing on other things)*												
3-factor “Pignon et al.”, 1-tier	3	Pooled (primary)	115.3	39	0.98	0.97	0.09 (0.07, 0.12)	154.8	39	0.96	0.95	0.13 (0.10, 0.15)
		PPS sample only	69.9	39	0.97	0.96	0.07 (0.03, 0.10)	105.4	39	0.93	0.90	0.12 (0.09, 0.14)
*Non-planned factor analysis based on 8 items (without items 4 [convinced symptoms serious]*,* 10 [doctor does not seriously]*,* and 12 [focusing on other things])*												
3-factor “Pignon et al.”, 1-tier	3	Pooled (primary)	56.8	17	0.99	0.99	0.08 (0.04, 0.12)	70.1	17	0.99	0.98	0.10 (0.07, 0.14)
		PPS sample only	41.5	17	0.98	0.96	0.08 (0.02, 0.12)	50.0	17	0.98	0.97	0.08 (0.03, 0.13)

The pooled sample included 354 participants, of whom 194 were clinical trial participants with persistent physical symptoms, and 160 healthy volunteers. All models were fitted using weighted least square mean and variance adjusted (WLSMV) estimation, with robust fit indices. A bifactor model with 3 local factors for the cognitive, affective, and behavioral aspects of symptom preoccupation was also evaluated but had solutions with negative variances (not tabulated). p.w.c., problems with convergence and/or Heywood cases; PPS, persistent physical symptoms.

^a^ Negative variance for item 4 (convinced symptoms serious) and the affective factor.

^b^ Negative variance for the affective factor.

#### SSD-12 internal consistency

As reported in Table 4, the SSD-12, SSD-8, and SSD-8 subscales exhibited excellent internal consistency in the pooled data, and adequate internal consistency in the PPS subsample. Results were similar for the SSD-12–1W and the SSD-8–1W.

#### SSD-12 correlations relevant for construct validity, including change scores

Correlations with other constructs are reported in Tables S7 and S8. As previously reported, correlations with the PHQ-15 were moderate and there was a strong correlation in change of the PHQ-15–1W and the SSD-12–1W. In the pooled data, correlations with general anxiety and depression were strong and moderate to strong respectively (*r* = 0.74, 0.46). Similar results were seen for the SSD-12–1W (Table S2).

#### SSD-12 test-retest reliability

The conventional SSD-12 was administered twice within 14.7 (SD = 0.9) days in the healthy volunteer data, where test-retest reliability was below target (ICC = 0.62, *r* = 0.63). The SSD-12–1W had unacceptable test-retest reliability in the healthy volunteer data (ICC = 0.36, *r* = 0.36), including as an average over two timepoints (ICC = 0.53). In the PPS data, measurements could only be compared over the first 16.3 (SD = 1.7) days of treatment. Despite the ongoing intervention, reliability appeared borderline acceptable (ICC = 0.64, *r* = 0.75), and good when averaged over two timepoints (ICC = 0.78). For details, see Table S9.

## Discussion

This study evaluated the measurement properties of the Patient Health Questionnaire 15 (PHQ-15), a widespread measure of somatic symptom burden, and the Somatic Symptom Disorder B-criteria scale 12 (SSD-12), a widespread measure of symptom preoccupation, focusing on Swedish conventional and 1-week versions. This was based on data from clinical trial participants with persistent physical symptoms and from healthy volunteers. The unique contribution of this study was that it systematically evaluated revised 1-week versions of both the PHQ-15 and SSD-12; two questionnaires that are widely considered for repeated joint measurements in behavioral medicine. In contrast with our hypotheses, we found that the PHQ-15 did not exhibit a clear-cut, stable, factor structure. This problem appeared unrelated to the choice of a 4- or 1-week timeframe, and appeared to stem at least partially from the pain and fatigue items bordering between forming either one common, or two distinct, local factors. The best-fitting model specified a merged *Pain/fatigue* factor, but with covariance over items 2 and 3, and the general factor explained a narrow majority of the variance, thus supporting the summing of the corresponding 11 items. Similar to the previous literature^6^, this could be modelled either as a hierarchical of bifactor structure. Also in contrast with our hypotheses, we found the SSD-12 to exhibit an unexpected 3-factor structure similar to that presented by Pignon et al. ^17^, with factors for *Expectation of a chronic course*, *Health anxiety*, and *Symptom focus and impairment*. Though these factors were highly correlated (rs = 0.85–0.94), a unifactorial solution did not achieve acceptable fit. Internal consistency and construct validity were mostly in line with expectations and supported for both the PHQ-15 and the SSD-12. Test-retest reliability was generally unsatisfactory in the healthy volunteer data. Worthy of particular caution, test-retest reliability of the SSD-12–1W in the non-clinical participants was poor, speaking against use of this questionnaire in healthy population settings. On the other hand, test-retest reliability appeared to be defendable in the clinical data, including in the case of the 1-week variants. For a structured overview of a priori targets and hypotheses in relation to the outcome, see Tables S1 and S2.

A general pattern in this study was that factor structures were less distinct, and correlations weaker between the clinical variables of interest in the subsamples compared to the primary, pooled, sample. We interpret this primarily as an expected effect of restriction of range, as both samples were selected based on eligibility criteria related to the variables of interest. For example, only two participants in the PPS sample (2/194 = 1%) had an SSD-12 score below 10.^44^ One way of interpreting this could be that when variance in a dimension (e.g., symptom preoccupation) is lost, the explanatory power of that dimension is diminished, and other sources of variance will have more impact on the factor structure.^45^.

The 3-factor solution presented for a 11-item subset of the PHQ-15 in this study is similar to solutions presented previously in the literature^46–48^, although 4 local factors are more common^6^. The exclusion of items 4 (menstrual problems) and 8 (fainting spells) is almost universal and, considering expected frequencies (menstruation often being a monthly phenomenon), logical when a 1-week timeframe is employed. Our finding that the PHQ-15–1W has relatively similar properties as the PHQ-15 is also in line with the previous literature^20^. One aspect supporting use of the PHQ-11 (or PHQ-11–1W) over the PHQ-15 (or PHQ-15–1W), is that, at least with a conventional timeframe, the average variance extracted by the general symptom burden factor was just above 50% which implies that a sum score could be better for capturing change as compared to a conventional sum based on all 15 items.

The 3-factor solution presented for an 8-item subset of the SSD-12 was similar to the study by Pignon et al.^17^ but is not typical of the literature, where a unifactorial solution for all 12 items is more commonly endorsed^8–17^. A typical secondary finding was, however, that item 7 (reassured by others) was especially weakly correlated with the rest of the original scale^11,13–17^. As was pointed out by Abasi et al.^13^, this is likely because item 7 is phrased as a negation and focuses on the behavior of others rather than of the respondent (*“Others tell me that my physical problems are not serious”*). Similar problems have been seen with such items forming part of health anxiety scales^49^. The factors here denoted *Expectation of a chronic course*, *Health anxiety*, and *Symptom focus and impairment* replicating over studies from different countries, and over Latin and Germanic languages, gives some support for their validity. Tentatively, it may be the case that SSD-12, when not unifactorial, could be at least as likely to break down into these 3 correlated types of symptom preoccupation, as it is to break down into separate “DSM-like” factors for cognition, affect, and behavior. We also note that highly similar factors have been identified in the shape of a Catastrophizing factor (similar to *Expectation of a chronic course* identified here) and a Symptom focusing factor (similar to *Symptom focus and impairment* identified here) in analysis of the Cognitive and Behavioral Responses to Symptoms Questionnaire (CBRQ)^50^, lending further support for the validity of these factor solutions. However, scoring the SSD-12 as one sum arguably remains a reasonable option considering the unifactorial solution found preferable in most studies, and high factor correlations seen here.

Especially in the pooled data, we note that the SSD-12 closely aligns with health anxiety as measured using the HAI-14 (Table S7, above the diagonal). In the clinical data, i.e., with a lower level of abstraction, there appears to be considerably more nuance in that especially the *Expectation of a chronic course* was more distinct from the rest of the SSD-8, and more so health anxiety (*r* = 0.18), though this is a tentative conclusion based on factor loadings from this specific study (Table S7, below the diagonal). This view of symptom preoccupation as encompassing, but being a broader construct than, health anxiety (fear of, or preoccupation with, having or developing a serious disease), is consistent with expectation. This broader focus may perhaps explain the slightly better discriminative ability of the SSD-12 as compared to health anxiety scales for the purpose of identifying somatic symptom and related disorders.^51^ If symptom preoccupation is understood as the tendency to which an individual responds strongly to, and engages in behaviors contingent on, somatic symptoms, there could be room for further broadening of a scale such as the SSD-12 for example with regard to emotions other than fear.^52^.

This study had several limitations. The pooled sample size was within a range usually considered acceptable for factor analysis^24–26^, but results, especially those pertaining to the relatively complex bifactor models, might have been more stable with a larger sample. The most promising factor structures were derived from non-planned analyses, highlighting the need for replication. Most participants were self-referred highly educated women, which limits generalizability. We also note that robust fit indices tend to be more conservative than conventional variants, and that less is known about their properties. We adhered to the most common practice of adopting widely cited rule-of-thumb criteria developed for corresponding conventional fit indices.^41^ This is a conservative approach, which should be kept in mind in the comparison with other studies. Lastly, in the comparison of conventional and 1-week questionnaire versions, a common motivation for employing repeated measurements is to improve reliability as a function of it being possible to include several measurements in the same analysis, e.g., to accurately place a regression line. In the present study, we saw clearly that even when reliability is questionable for a single measurement point, the reliability for the average of two measurement points may be considerably better (Table S9). Future studies could compare the reliability of measurement schemes as opposed to single measurements.

The main clinical implication of this study is that the PHQ-15 and SSD-12 may be administered in revised 1-week variants to patients with persistent physical symptoms. In the routine care environment this may be highly useful. By monitoring how somatic symptom burden and symptom preoccupation change on a weekly basis, the clinician may have an easier time initiating, tailoring, and evaluating interventions for example during psychotherapy. Also relevant for a meaningful overview of the patients symptom profile, this study strengthens the empirical evidence for scoring 8 items from the SSD-12 in terms of separate subscales of *Expectation of a chronic course*, *Health anxiety*, and *Symptom focus and impairment*. Although all three scales are correlated, for individual patients, there may be a need for intervention within one domain but not another, and change can be observed in the three domains separately; thereby facilitating clinical reasoning and decision making.

In summary, this study corroborates that the PHQ-15 can be conceptualized as having a hierarchical or bifactorial structure, while the SSD-12 appeared to have a 3-factor structure similar to that recently proposed by Pignon et al. ^17^. Solutions were similar when the timeframe was changed to concern the past week only. Tentatively, both scales appear to have redundant items and showed evidence of questionable reliability in healthy sample data. This study is cautiously supportive of the PHQ-15 and SSD-12 being administered with a revised 1-week focus to individuals with persistent physical symptoms if repeated measurements are employed.

## Supplementary Information

Below is the link to the electronic supplementary material.

Supplementary Material 1

## Data Availability

Individual participant data from this study fall under Swedish and European Union data protection and privacy legislation and can therefore not be shared freely. Requests for data or additional estimates may be directed to the corresponding author (EA) and will be considered on a case-by-case basis, in accordance with current legislation and local policies of the sponsor.
