# Supplementary material for: Measurement properties of the Patient Health Questionnaire 15 (PHQ-15) and Somatic Symptom Disorder B-criteria scale (SSD-12), including revised 1-week versions
Source: Sci Rep. 2026 Apr 26;16:13415. doi: 10.1038/s41598-026-50290-y (PMC13111608; doi:10.1038/s41598-026-50290-y)
Supplement: Supplementary file 1 — Supplementary Material 1 [file 41598_2026_50290_MOESM1_ESM.pdf]

Supplement for: Measurement properties of the Patient Health Questionnaire 15 (PHQ-15) and Somatic Symptom Disorder B-criteria scale (SSD-12), including revised 1-week versions

Jonna Hybelius; Sandra af Winklerfelt Hammarberg ; Alice Ahnlund Hoffmann; Edward Spansk; Anna Olsson; Emma Strand; Lina Söderström Winter; Tomas Åkerlund; Daniel Björkander; Amanda Kosic; Gabriel Chahin; Majken Epstein; Erland Axelsson

Supplement for: Measurement properties of the Patient Health Questionnaire 15 (PHQ-15) and Somatic Symptom Disorder B-criteria scale (SSD-12), including revised 1-week versions

Table S1. Patient Health Questionnaire 15 (PHQ-15) hypotheses/targets, preregistered in the Open Science Framework (https://osf.io/g7zdt)

**Outcome / domain**	**A priori hypothesis / target**	**Result for conventional version**	**Result for 1-week version**
Item-total correlations<br>*(See Table 2)*	All items except 4 (menstrual problems), 8 (fainting spells), and 11 (sexual problems) will exhibit adjusted item-total correlations (ITCs) ≥0.50.	Corroborated except for item 6 (chest pain; ITC=0.47) which was close to the target.	Corroborated except for items 2 (back pain; ITC=0.49) and 6 (chest pain; ITC=0.49) which were close.
Factor structure<br>*(See Tables 3, S3, S4)*	Approximation of bifactorial model presented by Witthöft and colleagues [1].	No, rather necessary to drop also items 2, 3, 5, and 11. Bifactor model with merged pain/fatigue domain factor could be fitted.	No, rather necessary to drop items 2, 3, 5, and 11. Bifactor model with merged pain/fatigue domain factor could be fitted, but not on the clinical subsample.
Internal consistency<br>*(See Table 5)*	Adequate internal consistency as indicated by an α and ω ≥0.70.<br><br>We expected an α of approximately 0.80.	Corroborated in the pooled sample (α=0.88), but variants lower in the clinical subsample.	Corroborated in the pooled sample (α=0.87), but variants lower in the clinical subsample.
Construct validity<br>*(See Tables S7, S8)*	Correlations with the SSD-12 of ca 0.30-0.60 in the clinical data, and 0.60-0.80 in the healthy volunteer data. Also, correlations in change scores in the clinical data to be in the same vicinity (0.30-0.60).<br><br>Correlations with general anxiety and depression around 0.30-0.65.	Clinical data: Corroborated, with r=0.30 correlation with the SSD-12 at baseline. Healthy volunteer data: No, rather r=0.35 at baseline.<br><br>Correlations with general anxiety and depression corroborated in the pooled data (r=0.64, 0.60), but lower in the subsamples.	Clinical data: Corroborated, with r=0.39 correlation with the SSD-12 at baseline, and r=0.52 correlation in change scores derived from fitted regression lines. Healthy volunteer data: No, rather r=0.34 at baseline.<br><br>Correlations with general anxiety and depression corroborated in the pooled data (r=0.66, 0.56), but lower in the subsamples.
Test-retest reliability<br>*(See Table S9)*	Approximately 2-week test-retest reliability Pearson r≥0.70, and an intraclass correlation coefficient (ICC) ≥0.50, ideally ≥0.70.	No, borderline ICC and Pearson r below target in healthy volunteer data. Average of two timepoints appears to be reliable.	No, not reliable in healthy volunteer data. Probably reliable in clinical data, and clearly so over two timepoints.
Impact on sum of a revised 1-week timeframe	We expected PHQ-15 sum to be approximately 25-40% higher when concerned with the past 4 weeks, as opposed to solely the past week [2]. *Note that this comparison was complicated by the two timeframe versions of the questionnaire only being administered during the same assessment for the healthy volunteers.*		In the healthy volunteer data, at the first timepoint, the PHQ-15 sum was 2.54 (SD=2.25) and the PHQ-15-1W sum was 2.27 (SD=2.05); a difference of 12%.

Table S2. Somatic Symptom Disorder B-criteria scale 12 (SSD-12) hypotheses/targets, preregistered in the Open Science Framework (https://osf.io/g7zdt)

**Outcome / domain**	**A priori hypothesis / target**	**Result for conventional version**	**Result for 1-week version**
Item-total correlations<br>*(See Table 2)*	All items except 7 (reassured by others) [3] will exhibit adjusted item-total correlations (ITCs) ≥0.50.	Corroborated except for item 7 (reassured by others; ITC=0.57) which was better than expected.	Corroborated except for item 7 (reassured by others; ITC=0.61) which was better than expected.
Factor structure<br>*(See Tables 4, S5, S6)*	Approximation of 3-factor model presented by Toussaint and colleagues [3].	No, rather similar to the 3-factor model proposed by Pignon and colleagues [4] and with improved fit without items 4, 10 and 12.	No, rather similar to the 3-factor model proposed by Pignon and colleagues [4] and with improved fit without items 4, 10 and 12.
Internal consistency<br>*(See Table 5)*	Adequate internal consistency as indicated by an α and ω ≥0.70.<br><br>We expected an α of approximately 0.85-0.90.	Corroborated and slightly better than expected (α=0.96).	Corroborated and slightly better than expected (α=0.96).
Construct validity<br>*(See Tables S7-S8)*	Correlations with the PHQ-15 of ca 0.30-0.60 in the clinical data, and 0.60-0.80 in the healthy volunteer data. Also, correlations in change scores in the clinical data to be in the same vicinity (0.30-0.60).<br><br>Correlations with general anxiety and depression around 0.40-0.70.	Clinical data: Corroborated, with r=0.30 correlation with the SSD-12 at baseline. Healthy volunteer data: No, rather r=0.35 at baseline.<br><br>Correlations with general anxiety and depression corroborated in the pooled data (r=0.74, 0.46), but lower in the healthy subsample.	Clinical data: Corroborated, with r=0.39 correlation with the SSD-12 at baseline, and r=0.52 correlation in change scores derived from fitted regression lines. Healthy volunteer data: No, rather r=0.34 at baseline.<br><br>Correlations with general anxiety and depression corroborated in the pooled data (r=0.77, 0.60), but lower in the healthy subsample.
Test-retest reliability<br>*(See Table S9)*	Approximately 2-week test-retest reliability Pearson r≥0.70, and an intraclass correlation coefficient (ICC) ≥0.50, ideally ≥0.70.	No, borderline ICC and Pearson r below target in healthy volunteer data. Average of two timepoints appears to be reliable.	No, not reliable in healthy volunteer data. Probably reliable in clinical data, and clearly so over two timepoints.

Supplement for: Measurement properties of the Patient Health Questionnaire 15 (PHQ-15) and Somatic Symptom Disorder B-criteria scale (SSD-12), including revised 1-week versions

PHQ-15 factor analysis (detailed description)

In planned analyses, the bifactor models had parameters exhibiting negative variance (Heywood cases), and we were not able to reconcile these problems through small, theoretically sound, changes informed by the modification indices. As is illustrated in Table 3, none of the planned factor analyses were indicative of acceptable fit. The unifactorial model performed the worst, and the remaining models fared about equally poorly.

Subsequent evaluation of the factor structure was exploratory, and did not follow a preregistered plan: Certain sources of misspecification were recurrent and easily recognized. Item 11 (sexual pain/problems) did not belong to the gastrointestinal factor and showed only modest correlations with other items overall. Surprisingly, item 5 (headaches) was also clearly implicated in the modification indices, and did not appear to clearly belong to any symptom domain factor, which prompted us to exclude this item. The exclusion of items 11 and 5 led to the 3-factor and 4-factor models having borderline acceptable fit on the remaining 11 items (Table 3). The 4-factor models, however, could not be consistently run on the clinical data, and their top modification indices provided little guidance for further optimization. Modification indices for the 3-factor model were clearly suggestive of excess local covariance over the only two remaining pain items of the pain/fatigue factor (items 2 and 3). With covariance over these two items, a 3-factor model achieved acceptable fit, both as a 2-tier and bifactor variant (Table 3). The local factors were: *Cardiopulmonary* (items 6, 7, 9, 10), *Gastrointestinal* (items 1, 12, 13), and *Pain/fatigue* (items 2, 3, 14, 15). For loadings, see Table S3. Item 14 (tired or low energy) had the highest proportion of variance explained by the general factor in both solutions (81.5-84.1% in the pooled data), and the highest loading subfactors were *Pain/fatigue* (87.8%) followed by *Cardiopulmonary* (76.7%). In the pooled data, the average variance extracted for the 2-tier variant was 66.3% (50.4% indirectly by the general factor), and the corresponding figure for the bifactor variant was 69.6% (50.1% by the general factor).

For the 1-week version, fit indices were similar but slightly less beneficial. The 4-factor 2-tier and 3-factor bifactor models could not be fitted as reliably. Factor loadings for the final 3-factor 2-tier model are listed in Table S4. Again, item 14 (tired or low energy) had the highest proportion of variance explained by the general factor (86.5% in the pooled data), and the highest loading subfactor was *Pain/fatigue* (97.2%) followed by *Cardiopulmonary* (89.3%). The average variance extracted was 63.1% (49.5% indirectly by the general factor). In the following text, the conventional PHQ-15 sum is referred to as "PHQ-15", the sum of the 11 items retained is referred to as "PHQ-11", and 1-week versions have the suffix "1W".

Supplement for: Measurement properties of the Patient Health Questionnaire 15 (PHQ-15) and Somatic Symptom Disorder B-criteria scale (SSD-12), including revised 1-week versions

Table S3. Standardized factor loadings from acceptable factor solutions for 11 items from the conventional PHQ-15 which focuses on the past 4 weeks

	**Pooled data, 3-factor 2-tier model, covariance over items 2 and 3**				**Pooled data, 3-factor bifactor model, covariance over items 2 and 3**	
**Item**	**1-tier loading**	**Factor**	**2-tier loading**		**Local loading**	**General loading**
1	0.89	Gastrointestinal	0.80		0.49	0.72
2	0.71	Pain/fatigue	0.94		0.12	0.69
3	0.63	Pain/fatigue	0.94		0.28	0.58
6	0.74	Cardiopulmonary	0.88		0.70	0.59
7	0.78	Cardiopulmonary	0.88		0.36	0.68
9	0.81	Cardiopulmonary	0.88		0.34	0.71
10	0.81	Cardiopulmonary	0.88		0.21	0.73
12	0.82	Gastrointestinal	0.80		0.52	0.64
13	0.96	Gastrointestinal	0.80		0.61	0.76
14	0.96	Pain/fatigue	0.94		0.18	0.92
15	0.79	Pain/fatigue	0.94		0.59	0.71

Table S4. Standardized factor loadings from an acceptable factor solution for 11 items from a revised PHQ-15 that focuses on the past week

	**Pooled data, 3-factor 2-tier model, covariance over items 2 and 3**		
**Item**	**1-tier loading**	**Factor**	**2-tier loading**
1	0.85	Gastrointestinal	0.71
2	0.59	Pain/fatigue	0.99
3	0.66	Pain/fatigue	0.99
6	0.71	Cardiopulmonary	0.95
7	0.76	Cardiopulmonary	0.95
9	0.76	Cardiopulmonary	0.95
10	0.81	Cardiopulmonary	0.95
12	0.9	Gastrointestinal	0.71
13	0.94	Gastrointestinal	0.71
14	0.94	Pain/fatigue	0.99
15	0.74	Pain/fatigue	0.99

SSD-12 factor analysis (detailed description)

As illustrated in Table 5, neither the most widespread 3-factor model nor the unifactorial model achieved adequate model fit based on robust fit indices and the prespecified criteria. Subsequent evaluation of the factor structure was exploratory, and did not follow a preregistered plan: Without item 7, which we dropped due to its problematic ITCs (see above), these models still did not achieve adequate fit. The recently proposed solution by Pignon and colleagues[4] fared considerably better, though the structure did not convincingly capture local dependencies over items 1 (symptoms sign of disease) and 4 (convinced symptoms serious), or 6 (preoccupied most of the day) and 12 (focusing on other things). Item 10 (doctor does not take seriously) also appeared to tap into two factors. Though, with local covariance, the model was borderline acceptable (Table 5), we also evaluated a version where we simply dropped item 4 (we preferred item 3 based on its distinct health anxiety as opposed to "chronic course" phrasing, see below), item 10 (to enable distinct subscales under simple sum scoring), and 12 (we preferred item 6 based on its distinct worry phrasing). Model fit was largely adequate, and relatively easy to interpret. The factors were: *Expectation of a chronic course* (items 8, 11), *Health anxiety* (items 1, 2, 5), and *Symptom focus and impairment* (items 3, 6, 9). Based on the pooled data, factor loadings for the final 3-factor model are listed in Table S5. The factor correlations were: *Expectation of a chronic course* vs *Health anxiety* 0.85, *Expectation of a chronic course* vs *Symptom focus and impairment* 0.92, and *Health anxiet*y vs *Symptom focus and impairment* 0.94. The average variance extracted was 87.6%.

In factor analysis of the revised 1-week version, fit indices were similar but slightly less favorable. Factor loadings from the pooled data are found in Table S6. In the following text, the conventional SSD-12 whole sum based on 12 items will be referred to as "SSD-12", the sum of the 8 items retained in the factor analysis will be referred to as "SSD-8", and the 1-week versions are given the suffix "1W".

Table S5. Standardized factor loadings from an acceptable factor solution for 8 items from the conventional SSD-12

	**Pooled data, 3-factor 1-tier model**	
**Item**	**1-tier loading**	**Factor**
1	0.83	Health anxiety
2	0.94	Health anxiety
3	0.93	Symptom focus and impairment
5	0.94	Health anxiety
6	0.92	Symptom focus and impairment
8	0.98	Expectation of a chronic course
9	0.96	Symptom focus and impairment
11	0.98	Expectation of a chronic course

Table S6. Standardized factor loadings from an acceptable factor solution for 8 items from a revised SSD-12 version that focuses on the past week

	**Pooled data, 3-factor 1-tier model**	
**Item**	**1-tier loading**	**Factor**
1	0.89	Health anxiety
2	0.98	Health anxiety
3	0.91	Symptom focus and impairment
5	0.96	Health anxiety
6	0.90	Symptom focus and impairment
8	0.99	Expectation of a chronic course
9	0.94	Symptom focus and impairment
11	0.98	Expectation of a chronic course

Table S7. Pearson correlations with other scales relevant for construct validity, conventional scale versions: pooled and clinical sample

	PHQ-15	PHQ-11	PHQ-11-CP	PHQ-11-GI	PHQ-11-P/F	SSD-12	SSD-8	SSD-8-EC	SSD-8-FI	SSD-8-HA	PHQ-2	GAD-7	HAI-14	WD2-12
PHQ-15	-	0.98*	0.80*	0.81*	0.86*	0.79*	0.79*	0.78*	0.75*	0.69*	0.60*	0.64*	0.69*	0.75*
PHQ-11	0.96*	-	0.80*	0.83*	0.88*	0.80*	0.81*	0.80*	0.77*	0.71*	0.60*	0.64*	0.70*	0.74*
PHQ-11-CP	0.67*	0.68*	-	0.51*	0.57*	0.66*	0.66*	0.58*	0.65*	0.62*	0.44*	0.58*	0.59*	0.61*
PHQ-11-GI	0.58*	0.62*	0.14*	-	0.57*	0.64*	0.64*	0.65*	0.60*	0.55*	0.49*	0.51*	0.56*	0.54*
PHQ-11-P/F	0.65*	0.69*	0.23*	0.11	-	0.71*	0.72*	0.75*	0.68*	0.61*	0.56*	0.53*	0.61*	0.70*
SSD-12	0.30*	0.29*	0.34*	0.08	0.16*	-	0.99*	0.91*	0.96*	0.93*	0.66*	0.74*	0.89*	0.72*
SSD-8	0.28*	0.29*	0.34*	0.06	0.19*	0.97*	-	0.92*	0.96*	0.93*	0.65*	0.74*	0.89*	0.72*
SSD-8-EC	0.22*	0.22*	0.08	0.08	0.26*	0.53*	0.58*	-	0.84*	0.76*	0.59*	0.63*	0.77*	0.67*
SSD-8-FI	0.25*	0.26*	0.35*	0.04	0.14	0.85*	0.89*	0.40*	-	0.85*	0.66*	0.73*	0.83*	0.73*
SSD-8-HA	0.21*	0.22*	0.31*	0.03	0.10	0.84*	0.84*	0.23*	0.61*	-	0.58*	0.71*	0.89*	0.62*
PHQ-2	0.33*	0.34*	0.17*	0.19*	0.30*	0.46*	0.46*	0.29*	0.46*	0.33*	-	0.62*	0.59*	0.69*
GAD-7	0.35*	0.33*	0.35*	0.18*	0.14	0.59*	0.58*	0.26*	0.53*	0.52*	0.44*	-	0.72*	0.61*
HAI-14	0.15*	0.13	0.22*	0.02	0.03	0.68*	0.66*	0.18*	0.51*	0.74*	0.33*	0.54*	-	0.63*
WD2-12	0.50*	0.46*	0.35*	0.12	0.44*	0.36*	0.34*	0.18*	0.39*	0.21*	0.54*	0.36*	0.20*	-

Correlations above the diagonal are derived from the pooled sample (N=354), and correlations below the diagonal are derived from the clinical subsample with persistent physical symptoms (n=194). Note that correlations between scale variants (PHQ-15 and PHQ-11, SSD-12 and SSD-8) are inflated because these sums were derived from the same data as opposed to separate questionnaires administered in parallel. HAI-14, 14-item Health Anxiety Inventory; PHQ-2, Patient Health Questionnaire 2 (core symptoms of depression); WD2-12, 12-item self-report World Health Organization Disability Assessment Schedule 2. PHQ-15/11, Patient Health Questionnaire 15 and 11-item version – subscales being: CP, cardiopulmonary symptoms; GI, gastrointestinal symptoms; P/F, pain and fatigue symptoms. SSD-12/8, Somatic Symptom Disorder B-criteria scale 12 and 8-item version – subscales being: EC, expectation of a chronic course; FI, symptom focus and impairment; HA, health anxiety.

* $p < 0.05$

Table S8. Pearson correlations with other scales relevant for construct validity, conventional scale versions: healthy sample

	PHQ-15	PHQ-11	PHQ-11-CP	PHQ-11-GI	PHQ-11-P/F	SSD-12	SSD-8	SSD-8-EC	SSD-8-FI	SSD-8-HA	PHQ-2	GAD-7	HAI-14	WD2-12
PHQ-15	-													
PHQ-11	0.95*	-												
PHQ-11-CP	0.59*	0.59*	-											
PHQ-11-GI	0.65*	0.67*	0.29*	-										
PHQ-11-P/F	0.73*	0.80*	0.22*	0.19*	-									
SSD-12	0.35*	0.38*	0.27*	0.17*	0.33*	-								
SSD-8	0.35*	0.37*	0.26*	0.19*	0.32*	0.96*	-							
SSD-8-EC	0.42*	0.46*	0.25*	0.22*	0.44*	0.77*	0.81*	-						
SSD-8-FI	0.13	0.14	0.04	0.03	0.18*	0.74*	0.72*	0.39*	-					
SSD-8-HA	0.25*	0.25*	0.28*	0.15	0.15	0.81*	0.87*	0.49*	0.51*	-				
PHQ-2	0.20*	0.19*	0.04	0.12	0.19*	0.29*	0.21*	0.26*	0.13	0.10	-			
GAD-7	0.22*	0.25*	0.14	0.02	0.32*	0.33*	0.31*	0.33*	0.24*	0.19*	0.44*	-		
HAI-14	0.27*	0.28*	0.20*	0.20*	0.20*	0.65*	0.67*	0.47*	0.57*	0.60*	0.17*	0.29*	-	
WD2-12	0.16*	0.17*	0.10	0.12	0.12	0.29*	0.26*	0.21*	0.30*	0.17*	0.17*	0.19*	0.33*	-

Correlations derived from the healthy sample (n=160). Note that correlations between scale variants (PHQ-15 and PHQ-11, SSD-12 and SSD-8) are inflated because these sums were derived from the same data as opposed to separate questionnaires administered in parallel. HAI-14, 14-item Health Anxiety Inventory; PHQ-2, Patient Health Questionnaire 2 (core symptoms of depression); WD2-12, 12-item self-report World Health Organization Disability Assessment Schedule 2. PHQ-15/11, Patient Health Questionnaire 15 and 11-item version – subscales being: CP, cardiopulmonary symptoms; GI, gastrointestinal symptoms; P/F, pain and fatigue symptoms. SSD-12/8, Somatic Symptom Disorder B-criteria scale 12 and 8-item version – subscales being: EC, expectation of a chronic course; FI, symptom focus and impairment; HA, health anxiety.

* $p < 0.05$

Supplement for: Measurement properties of the Patient Health Questionnaire 15 (PHQ-15) and Somatic Symptom Disorder B-criteria scale (SSD-12), including revised 1-week versions

Table S9. Test-retest reliability, where the persistent physical symptoms (PPS) sample conducted the second assessment during therapy

	PPS sample (n=180)			PPS sample, HLP participants only (n=73)			Healthy volunteers (n=160)					
	Over: M=16.3 (SD=1.7) days in therapy			Over: M=16.8 (SD=1.6) days in therapy			Over: M=14.7 (SD=0.9) days without intervention					
Scale	Revised 1-week version			Revised 1-week version			Conventional version			Revised 1-week version		
	ICC (95% CI)		r	ICC (95% CI)		r	ICC (95% CI)		r	ICC (95% CI)		r
	One timepoint	Average		One timepoint	Average		One timepoint	Average		One timepoint	Average	
PHQ-15	0.63 (0.45, 0.74)	0.77 (0.62, 0.85)	0.67	0.66 (0.44, 0.79)	0.79 (0.61, 0.88)	0.70	0.61 (0.49, 0.70)	0.76 (0.66, 0.83)	0.63	0.58 (0.47, 0.68)	0.74 (0.64, 0.81)	0.58
PHQ-11	0.65 (0.50, 0.75)	0.79 (0.67, 0.86)	0.69	0.69 (0.49, 0.81)	0.82 (0.66, 0.90)	0.73	0.57 (0.45, 0.67)	0.73 (0.62, 0.80)	0.60	0.60 (0.49, 0.69)	0.75 (0.66, 0.82)	0.60
PHQ-11-CP	0.68 (0.59, 0.76)	0.81 (0.74, 0.86)	0.70	0.65 (0.48, 0.77)	0.79 (0.65, 0.87)	0.68	0.55 (0.43, 0.65)	0.71 (0.60, 0.79)	0.56	0.54 (0.42, 0.64)	0.70 (0.59, 0.78)	0.54
PHQ-11-GI	0.64 (0.54, 0.72)	0.78 (0.70, 0.84)	0.65	0.73 (0.60, 0.82)	0.84 (0.75, 0.90)	0.73	0.55 (0.43, 0.65)	0.71 (0.60, 0.78)	0.57	0.42 (0.29, 0.54)	0.60 (0.45, 0.70)	0.42
PHQ-11-P/F	0.67 (0.54, 0.76)	0.80 (0.70, 0.86)	0.70	0.67 (0.48, 0.79)	0.80 (0.65, 0.88)	0.70	0.57 (0.45, 0.66)	0.72 (0.62, 0.80)	0.58	0.63 (0.53, 0.72)	0.77 (0.69, 0.83)	0.63
SSD-12	0.64 (0.21, 0.81)	0.78 (0.35, 0.90)	0.75	0.60 (0.22, 0.79)	0.75 (0.36, 0.88)	0.70	0.62 (0.52, 0.71)	0.77 (0.68, 0.83)	0.63	0.36 (0.22, 0.49)	0.53 (0.36, 0.66)	0.36
SSD-8	0.66 (0.29, 0.82)	0.80 (0.45, 0.90)	0.76	0.64 (0.25, 0.81)	0.78 (0.40, 0.90)	0.73	0.64 (0.54, 0.72)	0.78 (0.70, 0.84)	0.65	0.34 (0.19, 0.47)	0.50 (0.32, 0.64)	0.35
SSD-8-EC	0.53 (0.27, 0.69)	0.70 (0.43, 0.82)	0.61	0.56 (0.24, 0.74)	0.72 (0.39, 0.85)	0.65	0.67 (0.58, 0.75)	0.80 (0.73, 0.86)	0.67	0.58 (0.47, 0.67)	0.73 (0.64, 0.81)	0.59
SSD-8-FI	0.64 (0.43, 0.76)	0.78 (0.60, 0.86)	0.69	0.60 (0.39, 0.75)	0.75 (0.56, 0.85)	0.64	0.43 (0.29, 0.55)	0.60 (0.45, 0.71)	0.43	0.11 (-0.04, 0.26)	0.20 (-0.09, 0.42)	0.11
SSD-8-HA	0.70 (0.42, 0.83)	0.82 (0.59, 0.90)	0.77	0.63 (0.35, 0.78)	0.77 (0.52, 0.88)	0.69	0.56 (0.45, 0.66)	0.72 (0.62, 0.80)	0.58	0.16 (0.01, 0.31)	0.28 (0.02, 0.47)	0.17

While the analysis of test-retest reliability based on the healthy volunteer data was planned, the analysis of congruence over the baseline and week 2 measurements in the clinical data was not planned. Although these latter correlations calculated over two timepoints during therapy would be expected to be lower than correlations calculated over two timepoints outside of therapy (the typical focus of test-retest reliability evaluations), clinical estimates were amended as a frame of reference. HLP, healthy lifestyle promotion; ICC, two-way absolute-agreement random-effects intraclass correlation coefficient. PHQ-15/11, Patient Health Questionnaire 15 and 11-item version – subscales being: CP, cardiopulmonary symptoms; GI, gastrointestinal symptoms; P/F, pain and fatigue symptoms. SSD-12/8, Somatic Symptom Disorder B-criteria scale 12 and 8-item version – subscales being: EC, expectation of a chronic course; FI, symptom focus and impairment; HA, health anxiety.

References

1 Witthöft, M., Hiller, W., Loch, N. & Jasper, F. The latent structure of medically unexplained symptoms and its relation to functional somatic syndromes. *Int. J. Behav. Med.* **20**, 172-183 (2013). https://doi.org/10.1007/s12529-012-9237-2

2 Joustra, M. L., Janssens, K. A. M., Schenk, H. M. & Rosmalen, J. G. M. The four week time frame for somatic symptom questionnaires reflects subjective symptom burden best. *J. Psychosom. Res.* **104**, 16-21 (2018). https://doi.org/10.1016/j.jpsychores.2017.11.006

3 Toussaint, A. *et al.* Development and Validation of the Somatic Symptom Disorder-B Criteria Scale (SSD-12). *Psychosom. Med.* **78**, 5-12 (2016). https://doi.org/10.1097/psy.0000000000000240

4 Pignon, B. *et al.* Somatic Symptom Disorder-B criteria scale (SSD-12): Psychometric properties of the French version and associations with health outcomes in a population-based cross-sectional study. *J. Psychosom. Res.* **176**, 111556 (2024). https://doi.org/10.1016/j.jpsychores.2023.111556
